# The Football Players’ Health Study at Harvard University: Design and objectives

**DOI:** 10.1002/ajim.22991

**Published:** 2019-06-18

**Authors:** Ross Zafonte, Alvaro Pascual‐Leone, Aaron Baggish, Marc G. Weisskopf, Herman A. Taylor, Ann Connor, Jillian Baker, Sarah Cohan, Chelsea Valdivia, Theodore K. Courtney, I. Glenn Cohen, Frank E. Speizer, Lee M. Nadler

**Affiliations:** ^1^ Football Players Health Study at Harvard University, Harvard Medical School Boston Massachusetts; ^2^ Department of Physical Medicine and Rehabilitation, Spaulding Rehabilitation Hospital, Massachusetts General Hospital, Brigham and Women's Hospital Harvard Medical School Boston Massachusetts; ^3^ Berenson‐Allen Center and Division for Cognitive Neurology, Department of Neurology, Beth Israel Deaconess Medical Center Harvard Medical School Boston Massachusetts; ^4^ Cardiovascular Performance Program, Department of Medicine, Massachusetts General Hospital Harvard Medical School Boston Massachusetts; ^5^ Department of Environmental Health Harvard TH Chan School of Public Health Boston Massachusetts; ^6^ Department of Medicine, Cardiovascular Research Institute Morehouse Medical School Atlanta Georgia; ^7^ Harvard Law School Cambridge Massachusetts; ^8^ Channing Division of Network Medicine, Department of Medicine, Brigham and Women's Hospital Harvard Medical School Boston Massachusetts; ^9^ Dana Farber Cancer Center Harvard Medical School Boston Massachusetts

**Keywords:** brain, cardiac, football, health

## Abstract

The Football Players Health Study at Harvard University (FPHS) is a unique transdisciplinary, strategic initiative addressing the challenges of former players’ health after having participated in American style football (ASF). The whole player focused FPHS is designed to deepen understanding of the benefits and risks of participation in ASF, identify risks that are potentially reversible or preventable, and develop interventions or approaches to improve the health and wellbeing of former players. We are recruiting and following a cohort of former professional ASF players who played since 1960 (current *n* = 3785). At baseline, participants complete a self‐administered standardized questionnaire, including initial reporting of exposure history and physician‐diagnosed health conditions. Additional arms of the initiative are addressing targeted studies, including promising primary, secondary, and tertiary interventions; extensive in‐person clinical phenotyping, and legal and ethical concerns of the play. This paper describes the components of the FPHS studies undertaken and completed thus far, as well as those studies currently underway or planned for the near future. We present our initiatives herein as a potential paradigm of one way to proceed (acknowledging that it is not the only way). We share what we have learned so that it may be useful to others, particularly in regard to trying to make professional sports meet the needs of multiple stakeholders ranging from players to owners, to fans, and possibly even to parents making decisions for their children.

## 1 INTRODUCTION

Over the last 20 years, there has been increasing concern both about the acute injury effects as well as the long‐term consequences to athletes participating in high‐impact contact sports.^1^ These are not new concerns. Incidental case reports of acute traumatic injuries resulting in significant incapacitation and even deaths had been reported for over 50 years. More recently, chronic or late‐onset significant morbidity associated with participation in high‐impact sports has become of increasing concern, as reports of significant neurodegenerative diseases occurring in former prominent athletes, particularly related to those who played professional American style football (ASF), have made news in both the scientific as well as the lay press.^2^ Other chronic conditions, including musculoskeletal, cardiovascular, sleep disorders and behavioral mental health conditions have also been reported.^3^, ^4^ However, for the most part, the published literature falls short in providing sufficient data to make informed judgments to quantify the magnitude of the risks associated with ASF for any of these conditions. This has the unfortunate effect of placing a burden on former players, potential players, and their families, as well as other stakeholders to make potentially lifestyle and health‐related decisions without adequate facts. It should be noted that this manuscript is designed to describe a strategic programmatic response to a research need and a series of studies under a large umbrella rather than a single study.

In an effort to better document the potential long‐term consequences of participation in ASF, the National Football League Players Association (NFLPA) in 2014 put forward a nationally advertised major Request for Proposals to study the health and welfare status of retired professional ASF players. The proposal asked for studies to assess and develop potential new preventive, diagnostic, and therapeutic interventions that would mitigate potential long‐term consequences of participation in the sport. In response, Harvard University developed the Football Players Health Study (FPHS), which was designed as a multidisciplinary investigative team approach to address these issues. This effort was formally funded in 2014***.*** The goal of the Football Players Health Study at Harvard University (FPHS) is to further understand the benefits and risks of participation in ASF, identify those risks that are potentially reversible or preventable, and develop interventions or approaches to improve the broad array of issues impacting the health and wellbeing of former ASF players.

From the onset, it was clear that the success of this program would be dependent on understanding and being committed to the concept of ongoing engagement with the population of interest in a participatory approach throughout the research process.^5^ We initially conducted a number of focused meetings with representatives of the NFLPA as well as former ASF players from a variety of other player associations. These sessions provided input into prioritizing clinically meaningful targets for assessment, intervention, and potential functional improvements. Follow‐up meetings led to working groups of Harvard University faculty who came together to design a comprehensive set of studies, as indicated below, around the theme of “the whole player, the whole life.” Issues considered included, but were not limited to, identifying factors that could mitigate risk of having an injury; understanding consequences of injury as well as other factors associated with participating in the sport at the professional level on short and long‐term health impacts; and, to the degree possible, understanding the long‐term consequences for both physical and social impacts of having participated in the sport. In addition, we proposed to explore potential new approaches to therapeutics to lessen long‐term consequences of the unique exposures and putative injuries to which players are exposed. Not the least of our objectives was to determine the magnitude of the risk rates of a wide variety of outcomes. Such data would give all stakeholders better estimates for making potentially life‐changing decisions regarding participating in ASF. Because of the complex nature of player‐team relationships, a group of bioethics and legal scholars formed an additional unique component of our studies to explore and address the ethical and legal implications of the way professional ASF is organized. Finally, a significant component of our efforts is to keep the former players informed of our progress. This has been done through both a series of former player advisor group meetings and social media efforts to both inform former players on the progress of the studies and encourage participation in the ongoing efforts.

This paper describes the components of the FPHS studies undertaken and completed thus far, as well as those studies currently underway or planned for the near future. We present our initiatives herein as a potential paradigm of one way to proceed. We fully acknowledge that our approach is not the only way, but believe that what we have learned may be useful to others, particularly in regard to trying to make professional sports meet the needs of multiple stakeholders ranging from players to owners, to fans, and possibly even to parents making decisions for their children.

## 2 SCOPE OF THE STUDIES

We initially established two important Advisory groups. The first was made up of former NFL players who represented a spectrum of regional areas of the US, positions played, and different age groups. These former players provided essential insight into the concerns and questions that were most germane to the former player groups. The second was a group of local physician/scientists representing the range of research domains believed to be important to consider. Both groups have continued to evaluate and provide input into the research designs undertaken.

**Figure 1:**
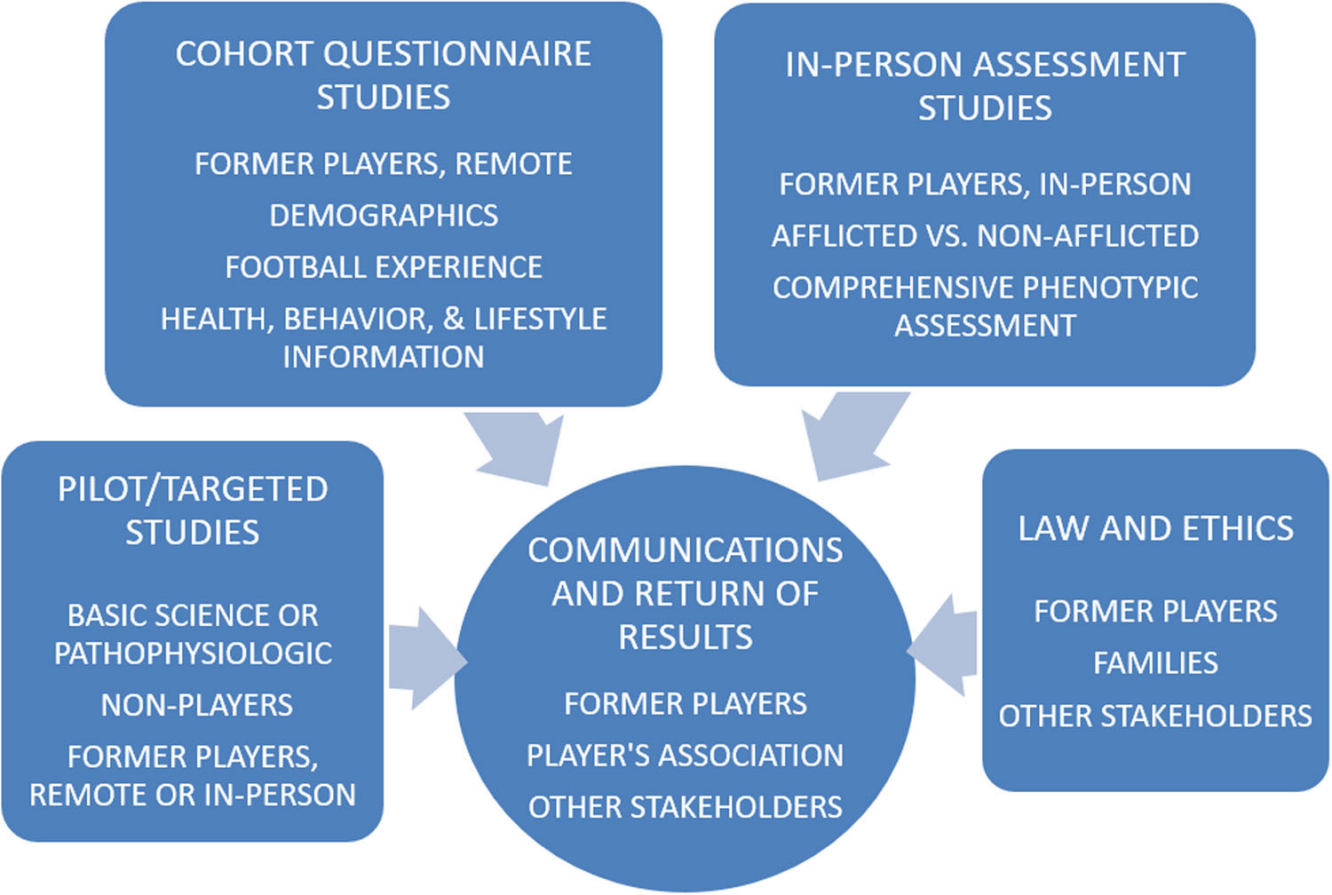
Range of studies undertaken, ongoing, and planned [Color figure can be viewed at wileyonlinelibrary.com]

The range of studies can be divided into three broad categories (Figure 1). Within each of these categories, there are a number of substudies, some of which have been completed, some which are ongoing, and some which are still in the planning or early implementation stages. In addition, an important component is communication and return of results to the participating former players and other stakeholders.

### 2.1 Former player studies

A major effort of the FPHS focuses on studies of former NFL players with the goal of assessing risk factors associated with participating in professional ASF and the putative long‐term consequences to their health and wellbeing. This plan requires a coordinated set of studies. Initially, we needed to assess components related to the exposures that are or have been a necessary part of the game. Further, we are attempting to quantitate the current physical, social, and neurocognitive state of former players, and testing within nested case‐control subgroups newer diagnostic techniques and remote assessment tools. Eventually, we would hope to introduce potential new therapeutic modalities that may enhance the lives of former players after they leave the game.

#### 2.1.1 Cohort questionnaire studies

Beginning in 2014, we sought to enlist the participation of an as large as possible cohort of former ASF players who had participated in the NFL (or former American Football League). Our criterion for enrollment was “formerly played professional football at any point from 1960 to present.” “Formerly played” was defined as having received compensation as a player from an NFL team. The year 1960 was chosen because by that time the transition from the soft, leather helmet to the hard, plastic helmet had been established throughout the league. The eligibility to join the cohort is a dynamic one in which younger players are invited and encouraged to enroll as they declare themselves retired. In addition, as subsequent substudies identify former players who had not enrolled in the initial round of cohort data collection, they are invited to provide baseline data.

To determine the topics to include in the initial standardized questionnaire, we held focus group meetings with both former players and research advisors. We first identified the parameters that would permit us to measure some of the characteristics of “exposure” in professional football (eg, position played, years of play, nature of some of the injuries during active playing years, essential demographics, etc). We also identified a number of health‐related domains of concern, for which we believed, by using well‐validated questions, we could establish baseline health status for the proposed cohort. Because a significant portion of the eligible cohort had either a home address or an email address, but not both, we needed to assess the potential difference in response patterns that might occur using one rather than both methods for contact. We selected approximately 500 former players at random who had both home addresses and emails to assess the response rate and degree of completion of the various components of the questionnaire. We determined that the response patterns and degree of completeness were no different between administering the questionnaire by email (REDCap^C^) vs Scantron^c^ paper questionnaires, and thus both methods were used for those for whom we had appropriate contact information.

**Figure 2:**
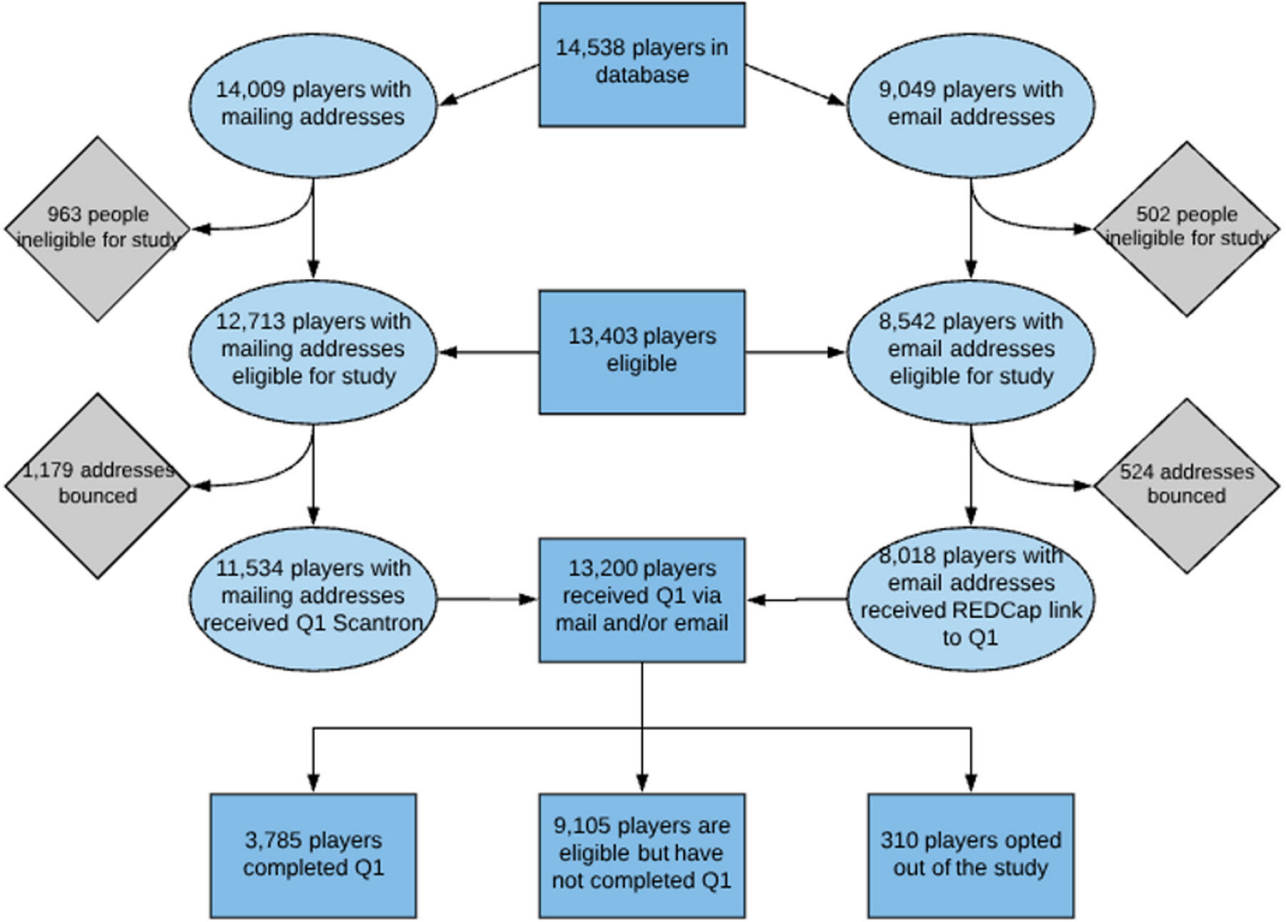
Flow chart of former player contacts [Color figure can be viewed at wileyonlinelibrary.com]

The original main sources for defining former players were lists provided by the NFL Players Association, supplemented by NFL Profootball Reference.^1^ Additional sources, many overlapping, included a number of philanthropic associations formed by former player groups, wives of current and former players, and other regional and local groups. These groups were asked to communicate with their members and to inform them of the study.^2^ Figure 2 describes the sources and number of former players for whom we initially believed we had obtained a contact address. Initially, we estimated that approximately 20 000 individuals played for one or more of the approximately 30 teams over the years starting in 1960. Of these, we estimated that approximately 4000 had died before the beginning of the follow‐up period. In February 2015, at the time of our first effort to contact the former players, and over the initial 3 years of follow‐up, we were able to confirm 14 538 individuals who met the criteria as former active players. Other members of the initially constructed lists had included coaches, management staff, and others who were not active players. We were able to confirm anticipated valid home addresses for approximately 12 713 players. In addition, we had available potential email addresses for 8542. Using combined mailings for both paper questionnaires and web‐based methods, we estimated that 13 403 former players with appropriate years played eligibility received our questionnaire in one or both forms (only the first method used to respond was counted). At present, the cohort is made up of 3785 former players who have completed our initial questionnaire. Newly retired players are continuing to enroll and plans exist to follow them over time.

#### 2.1.2 Content and purpose

Our standardized questionnaire captures demographic and football exposure information that allows us to characterize the former players by age, race, football experience, and other usual characteristics of behavior related to lifestyle and general risk factors for injury and illness associated with participating during their playing years as well as postplaying years. In addition, we use well‐established questions to assess outcomes in a number of health domains.^3^ Other illnesses, surgeries, and medical conditions since retirement, which are self‐reported as confirmed by health practitioners, also are collected.

The data obtained from the cohort by questionnaire serves two essential purposes. The initially obtained data provides cross‐sectional estimates of the frequency of occurrence of health outcomes in a variety of domains. Along with these questions on outcomes, we are obtaining detailed information on exposure in organized football as well as general lifetime risk and behaviors that may affect these outcomes.

#### 2.1.3 Limitations

The overall response rate for the entire eligible cohort to date is approximately 30%. This is not dissimilar to other pro athlete cohorts.^6^ In selected exposure groups, we have found response rates by important demographic characteristics such as age, position played, and the number of seasons to be relatively comparable to the overall eligible population. In addition, although we can identify the prevalence of different health outcomes in our baseline data, the possibility of bias in estimating rates of these conditions among all former ASF players must be considered if players with certain conditions were more or less likely to join our cohort than those without the conditions. Importantly, though, this is not as problematic for studies of exposure‐health outcome associations with the cohort data, as such outcome‐dependent (or exposure‐dependent) participation or nonresponse does not create spurious associations between an exposure and outcome if no true association exists.^7^

The prospective component of the population cohort study began in January of 2019, with follow‐up questionnaires sent to all former players who previously responded, and with newly reported medical conditions documented by follow‐up to the players’ health care providers. The relation between exposures identified at baseline and the occurrence of new outcomes after baseline identified by such cohort follow‐up is much less susceptible to any biases related to participation in the baseline questionnaire. In addition, those former players who did not respond to the initial questionnaire will be given another opportunity to join the study. A few remote‐based substudies among the cohort members have been undertaken, either based upon the initial questionnaire responses or de novo ancillary hypotheses that are currently underway and are described in detail under the Targeted Studies section below.

### 2.2 In‐person assessment studies

An additional purpose of the cohort baseline questionnaire was to use the collected data on health status to identify former players who self‐reported to be either “afflicted” or “nonafflicted” in four important health‐related domains. A limited number of these participants, in matched groups by age and race, have been selected to be invited to come to Boston to undergo detailed specialized testing using both best‐available technology and some novel or exploratory technology to assess how self‐reported status in the domains of neurocognitive, cardiac, musculoskeletal pain, and sleep relate to objective measures in each of these domains. The first objective will use a multimodality protocol designed to provide a comprehensive phenotypic assessment of former players, with an emphasis on clarifying the link between subjective complaints or prior diagnoses (or lack thereof) identified on the questionnaire and gold‐standard objective assessments of disease status. Depending on the observed correlations, these objective measures, although in relatively in small numbers of subjects, can be used to test several hypotheses related to exposure in football by using nested case‐control designs.

For those participants who come to Boston for the in‐person assessments (IPA), we have developed a medical navigation system. At the end of the session, each subject who participates in the IPA will meet with an appropriate study team member to have the opportunity to discuss all clinical findings. Follow‐up for any incidental, clinically relevant findings will be recommended. When appropriate and desired, the subject will be helped with referral to clinical care facilities in his local community. This medical navigation also serves as an educational and guidance program geared toward the specific needs of participants across the study.

### 2.3 Targeted studies

While the development of the cohort of former players was being established, a number of opportunities to investigate a wide variety of issues related to the nature of the kinds of injuries and potential long‐term outcomes were identified through a local process of request for proposals among all of the Harvard institutions. These targeted studies are funded from the original grant proposal award. These were initially identified as Pilot Studies and more recently characterized as Targeted Studies. These studies take advantage of local ongoing work in the Harvard community by requesting research proposals for modest efforts that would provide data that could lead to substantial outside funding to expand the research and/or have significance to the former ASF player population. The proposals are competitively evaluated and considered for 1‐ to 2‐year funding periods. The criteria for funding are whether the study is considered innovative, feasible, and achievable in a limited funding period, considering the potential roadblocks to success and whether the proposers had the requisite skills and experience to conduct the research. These criteria are rated competitively by external, independent, domain‐specific experts, and funding decisions are made by the study leadership based on evaluation scores and programmatic needs. The studies are divided into basic science or pathophysiologic studies in both nonhuman and human (though not necessarily football player) populations, and studies in both active and retired ASF players. The targeted studies are each summarized by domains and described briefly in Table 1.

**Table 1:** Targeted Studies

Completed Studies			
Title	Principal investigator	Specific aims	Status
*Cardiovascular/Metabolic*			
Concentric left ventricular hypertrophy (LVH) among collegiate football athletes	Baggish (MGH)	To assess cardiac physiology and anatomy including LVH among collegiate football athletes before and following play seasons	Completed, Lin et al,^8^ Preliminary results used for successful NIH R01 grant
*General health*			
Risk communication about the injury in football	Viswanath (DFCI)	To assess current communication practices by health care providers working with athletes and risk‐related attitudes, perceptions, and communication preferences by athletes	Completed, Kroshus et al^9^and Baugh et al^10^
*Neurocognition*			
Brain trauma profiles associated with player positions in NFL	Hoshizaki (Univ of Ottawa)	To reconstruct impact situations from game films to understand the character and magnitude of brain trauma and to create an exposure matrix for each position	Completed, manuscript in preparation
Antibody therapy for treating brain injury and chronic traumatic encephalopathy	Lu (BIDMC)	To humanize a murine cis P‐tau antibody that effectively stops brain damage and fully prevents CTE after rmTBI in animal models, an essential step before starting clinical trials on TBI patients in 4 y	Completed, Kondo et al^11^and Albayram et al^12^; Stimulated future collaborations (see below)
	Mannix (BCH)		
Innovative new drug for traumatic brain injury (TBI) and chronic traumatic encephalopathy (CTE)	Lu (BIDMC)	To humanize a murine cis P‐tau neutralizing antibody that stops brain damage and fully prevents CTE after repeated mild TBI	Funding extended through August 2019, final report forthcoming
	Xiao Zhou (BIDMC)		
Swine model of concussion	Mannix (BCH)	To develop a clinically relevant closed head injury model of concussion to evaluate the correlation of in vivo tau PET with tau histopathology	Completed, manuscript in preparation
	El Fakhri (MGH)		
Traumatic brain injury (TBI) models in mice	Mannix (BCH)	A Knowledge Accelerator to bring together experts on TBI animal models, to generate critical novel insights that can bridge the gap between model systems and human studies, identify valuable diagnostic biomarkers for human disease, and provide reliable high throughput assays	Completed, manuscript in preparation
	Whalen (MGH)		
Transcranial light‐emitting diode (LED) therapy for the treatment of concussive brain injury	Meehan (BCH)	To determine the effect of treatment with red/near‐infrared LEDs on cognitive symptoms and quantitative measurements of cognitive function in patients suffering from chronic concussive brain injury	Completed, manuscript in preparation
On‐field brain movement and activity monitoring	Strangman (MGH)	To validate the use of NINscan for non‐invasively measuring brain movements within the skull, and quantify the relationship between head acceleration/deceleration and measurements of brain motion and physiology during common football activities	Completed, Strangman et al^13^
Low‐level near‐infrared laser light to reduce cognitive sequelae of repeated traumatic brain injury (TBI) in mice	Whalen (MGH)	To identify mechanistic insights into the potential beneficial effects of noninvasive light therapy on recovery from concussion, and lasting cognitive deficits after repeated TBI	Completed, Buckley et al^14^
Treatment of the persistent postconcussion syndrome with transcranial light‐emitting diodes (LED)	Zafonte (SRH/MGH)	To assess whether transcranial, high‐intensity LED applied outside the skull can improve frontal lobe function and working memory in patients with persistent postconcussion syndrome 6 months following injury	Completed, Iverson et al^15^; Further analyses and manuscripts in preparation
*Pain/Musculoskeletal*			
Blocking extracellular galectin‐3 in patients with osteoarthritis (modified citrus pectin trial)	Huang (MGH)	To evaluate whether modified citrus pectin is effective as a therapy for the signs and symptoms of osteoarthritis	Completed, manuscript in preparation
	Fisher (MGH)		
Protect when needed knee‐bracing technology	Walsh (Wyss)	To develop a novel, adaptive device that will offer maximal protection to the knee when forces are such that ligament integrity (particularly the ACL) is endangered, but otherwise represents no limitation to movement	Completed
	Kiapour (BCH)		
Sleep‐related health			
Cold fluid injections for the treatment of obstructive sleep apnea (OSA) (mouse model)	Anderson (MGH)	To demonstrate the safety and efficacy of using injectable cold fluid to selectively target and remove OSA‐associated fat deposits in the neck and upper airway	Completed, manuscript in preparation

Studies ongoing or in development:			
Title	Principal investigator	Specific aims	Status
*Multidimensional*			
Personal networks of former NFL players and the association with functional, cardiac, and cognitive outcomes	Dhand (BWH)	To use a web‐based survey to assess the relationship of personal networks with cognitive, cardiac, and functional outcomes	Launched November 2018
	Barabasi (NEU)		
	Pascual‐Leone (BIDMC)		
*Neurocognition*			
Brain health study	Germine (McLean)	To administer a web‐based assessment of the cognitive and psychological health of former NFL players, and provide an interpretation of results for each player	Completed, data undergoing analysis
	Pascual‐Leone (BIDMC)		
A Prospective controlled treatment trial for posttraumatic headaches (PTH)	Lebel (BCH)	To perform a sequential prospective controlled treatment trial on the efficacy of medications and minimally invasive nerve block interventions for PTH and neck pain in patients aged 16‐35 y	Extended through June 2019
	Stillman (BIDMC)		
*Pain/musculoskeletal*			
An inflammation responsive hydrogel depot for on‐demand drug delivery in the treatment of posttraumatic osteoarthritis (PTOA) in football players	Karp (BWH)	To develop an intra‐articular, self‐titrating drug delivery system based on hydrogels that can stably encapsulate disease‐modifying small molecule inhibitors of proteinases at high loading, and release on‐demand in response to changes in the traumatized joint.	Phase I complete, Joshi et al^16^; Phase II funded through July 2019
	Ermann (BWH)		
Kinematic analysis of teamstudy, standing and walking activities	Manor (HSL)	To determine the feasibility of conducting portable, in‐person kinematic assessments of gait and posture, obtained from standing and walking under single‐ and dual‐task conditions	Phase I complete, Manor et al^17^; Phase II complete, manuscript in preparation
Bridge‐enhanced ACL repair (BEAR)	Murray (BCH)	To perform a prospective, randomized controlled trial of 100 subjects to compare the BEAR technique to the gold standard of ACL reconstruction, with repeated MRIs at two years to assess if the early radiographic signs of posttraumatic osteoarthritis are different in the two groups	Phases I & II complete
			Murray et al,^18^ Murray et al^19^; Phase III funded through August 2019
Goal‐directed resilience training (GRIT) to mitigate chronic pain in former football players	Taylor (MSM)	To test the efficacy of a resilience skills training intervention for benefits for those at high risk	Protocol in development, study initiation in 2019
	Zafonte (MGH)		
*Sleep‐related health*			
Developing a scalable sleep health intervention to improve pain, quality of life, and health in football players	Bertisch (BWH)	To adapt a brief web‐based, sleep health intervention for players, and to evaluate its impact on sleep health, and through this on pain and other health‐related outcomes	Study began recruitment March 2019
	Redline (BWH)		

Abbreviations: BCH, Boston Children's Hospital; BU, Boston University; BIDMC, Beth Israel Deaconess Medical Center; BWH, Brigham and Women's Hospital; DFCI, Dana‐Farber Cancer Institute; HSL, Hebrew Senior Life Institute for Aging Research; McLean, McLean Psychiatric Hospital ; MGH, Mass General Hospital; MSM, Morehouse School of Medicine; NEU, Northeastern University; Wyss, Wyss Institute at Harvard.

A wide variety of Targeted Studies were developed over the initial 3 years of the study (Table 1). These include very basic immunologic assessments of impacts resulting from an acute injury, outcomes related to repeated mild traumatic brain injury in animals, cardiac assessment in active and retired players, video analysis of the biomechanics of exposure and the development of mechanical preventive strategies for stress to the knee during active exercise, among others. Similar additional basic and applied studies that are currently underway and being planned are also included in Table 1.

More recently, we have begun to develop remote assessment and intervention studies.

Because of the practical limitations of bringing large numbers of former players to Boston, it is clear that to the degree we can obtain standardized data remotely from former players living across the country we can increase our power to test a variety of hypotheses. Several such attempts are already underway. For example, using a smart phone‐based application specifically developed for the FPHS, we conducted a remote study of a neurological function using the effects of dual tasking on measures of balance (The Team Study).^17^ Participants who downloaded our Team Study app^4^ provided repeated measures over several weeks of balance and walking while doing mental arithmetic, with the data being transmitted remotely. Assessments of the results of this effort are currently underway. A second example is the Brain Health Study that uses a standardized series of cognitive assessment tools, remotely administered on an encrypted website, providing detailed assessments in several brain function domains. At the end of the procedures, the results are compared to a large standardized database, and we are able to provide individual participants with a personalized assessment of their cognitive function and styles. To date, 349 former ASF players have completed this assessment.

Other remote cohort substudies are currently just getting underway or are in the planning stages. These include the development of a scalable sleep intervention program to improve pain, quality of life, and health in former players; a goal‐directed resilience training study to mitigate chronic pain in a group of players living in the greater Atlanta area; and a study of personal networks with the potential to inform the development of tools to enhance health‐positive networking.

As a result of the initiation of a detailed follow‐up questionnaire study of all responders to the first round of baseline questionnaires, starting in 2019, we are collecting prospective incidence data over a 4‐year period in the established cohort and have increased opportunities for further remote studies in selected subgroups of the population. Those former players who did not respond to the initial questionnaire will be given another opportunity to join the study.

The basic population we are studying is largely a public and relatively easily identifiable cohort of former players. Therefore, one of the unique challenges of the study is to maintain the confidentiality of all medical information being gathered. Essential to gaining the trust of the former players, every possible effort to protect the security and confidentially of all health‐related data provided is made. To this end, we secured a Certificate of Confidentially from the National Institute of Health for each individual research protocol developed which includes former player participants. The Certificate prohibits disclosure of identifiable, sensitive research information to anyone not connected to the research (with certain exceptions; see https://humansubjects.nih.gov/coc/index). In addition, all data obtained are held in secure, custom‐built data repositories. All identifiable data are coded and removed from any working files. Access to identifying data is on a need‐to‐know basis, and only by specifically trained and vetted personnel.

### 2.4 Law and ethics initiative

At the onset of this effort, we recognized that because of the nature of the potential competing stakeholders’ interests in the NFL (owner, players, agents, physicians, families, fans, etc.), the interactions of these stakeholders raised myriad potentially complex legal and ethical considerations. Our Law and Ethics team's first task was to determine not only who these stakeholders were, but also to map the nature of their interactions. This effort encompassed a variety of distinct projects with the primary goal of understanding the legal and ethical issues that may enhance or impede players’ health and welfare. In keeping with the mantra, “the whole player, the whole lifetime,” this component of the study examined issues at various points of a player's lifetime—from competing for a spot in the NFL Combine, to active years of play, to retirement planning, and the way players and family members dealt with health issues after their playing years were over.

A series of studies were undertaken to assess how stakeholders’ perspectives interact (Table 2). We identified who the stakeholders in player health were, evaluated their legal and ethical obligations, and assessed the current successes as well as gaps and opportunities for each stakeholder in protecting and promoting player health.^20^, ^21^ In addition, we applied a series of legal and ethical principles to arrive at recommendations for positive change where needed.^22^ A second effort compared the NFL's policies and practices to those in place in Major League Baseball, the National Basketball Association, the National Hockey League, the Canadian Football League, and Major League Soccer, to assess best practices and make recommendations for areas deemed in need of improvement.^23^ In addition to these major reports, a series of additional studies on the legal and ethical aspects of the game were carried out over the first 3 years of the study. These include an examination of team doctors’ conflicts of interest and the ethical management thereof, an examination of the applicability of the Americans with Disabilities Act and the Genetic Information Nondiscrimination Act to NFL football and the NFL Combine, and an analysis of the applicability of workplace safety laws and guidelines (such as the Occupational Safety and Health Agency) to professional football. We also conducted a qualitative assessment project with one‐on‐one interviews of approximately 50 current and former players and another 50 of their family members. The output of this study is ongoing. The goal was to better understand the perspectives of these key stakeholders on the following topics: overall professional football experience, improving player safety, health, family, and social issues, support as a professional athlete, life after football, risk disclosure, and risk‐taking, health care and club medical staff, medical screenings, and injury and pain management. These data produced a theoretical evaluation of the path that a hypothetical college football player might face, from a legal and ethical perspective, in trying to enter the sport.^24^

**Table 2:** Law and ethics studies

Title	Principal investigator^a^	Specific aims	Status
Protecting and promoting the health of NFL players: legal and ethical analysis and recommendations	Cohen (Petrie Ctr)	Identified stakeholders, analyzed their legal and ethical obligations, and evaluate current successes, and gaps and opportunities for each stake‐holder. Applied a series of legal and ethical principles to arrive at recommendations for positive change.	Completed, Deubert et al^20^
	Lynch (Petrie Ctr)		
Proposal to address NFL Club doctors’ conflicts of interest and to promote player trust	Cohen (Petrie Ctr)	Evaluate the current structure of NFL player health care, in which club medical staff provide services to both the club and players.	Completed, Cohen et al^22^
	Lynch (Petrie Ctr)		
Evaluating NFL player health and performance: legal and ethical issues	Roberts (Houston)	Examine how the legal requirements of the Americans with Disabilities Act and Genetic Information Nondiscrimination Act interact with the evaluation of prospective players at the Combine and NFL players throughout their career.	Completed, Roberts et al^23^
	Cohen (Petrie Ctr)		
	Lynch (Petrie Ctr)		
Comparing Health‐related policies and practices in sports: the NFL and other professional leagues	Cohen (Petrie Ctr)	Examine the policies and practices of the NFL that concern player health, and compare them to those of other major professional sports leagues.	Completed, Deubert et al^21^
	Lynch (Petrie Ctr)		
Qualitative study/listening tour	McGraw (Hastings Ctr)	To better understand the perspectives of former players and family members of former players on overall NFL experience. Issues included were improving player safety, health, family, and social issues, support as a professional athlete, life after football, risk disclosure and risk‐taking, health care provided, medical screenings, and, injury and pain management.	Completed, McGraw et al^24^
	Cohen (Petrie Ctr)		
	Lynch (Petrie Ctr)		

^a^ Institutional abbreviations: Petrie Center, Harvard Law School; Houston, Houston Law School; Hastings Ctr, University of California, Hastings Law School.

### 2.5 Communications and return of results

A significant effort to inform the former ASF player community about the overall study and to, when possible, provide them with updates that relate specifically to their concerns in specific domains, is being carried out. To date, these efforts have focused on a variety of digital media pathways including emails; social media such as Twitter, Facebook, and LinkedIn; informational videos on the Study website; and status reports. Some of the remote player studies that are web‐based provide opportunities for rapid feedback of results to the participants (eg, Brain Health Study, Personal Networks Study). In addition, we have developed a process for providing contextual information from subject matter experts to not only interpret individual results appropriately but also to potentially offer suggestions to improve the participants’ daily lives and health outcomes. Similar approaches apply to results return for other emerging cohort analyses which are positioned within the framework of and providing motivation for, the player taking a proactive approach to their health. In addition, player advisors meet with investigators approximately once per year in person and participate in regular telephone conference calls to both discuss results and to communicate player issues of which they have become aware.

## 3 DISCUSSION

To date, we have established the largest ongoing study of living ASF former players. Because we continue to recruit both medium‐term and long‐term former players, as well as newly retired players, we anticipate that the cohort will grow in both size and significance as we move forward over the years. The current overall response rate to our questionnaire is approximately 28% across all position player groups. However, with regard to the distribution of responses within this group across age, years played, and positions played, our responders are similar compared to the entire potential cohort of former players who have not yet responded (Table 3). While there are limited studies for comparison among professional, team sport athlete populations, a recently reported study in a cohort of professional rugby players, with a response rate of 28%, is consistent with our own results.^6^

**Table 3:** Cohort responders with data in NFL Pro‐Reference (PFR) database compared to non‐responders in the database (preliminary assessment as of September 2018)

	Q1 responders, PFR data	Q1 non‐responders, PFR data
*N*	3099^a^	8555
Age, mean (SD), y	56.9 (13.5)^b^	51.1 (12.3)^b^
Playing, mean (SD), wt	233.0 (38.3)	229.7 (39.8)
Height, mean (SD), inches	74.2 (2.3)	73.7 (2.5)
Years played, y	6.0 (3.6)^c^	5.2 (3.5)^c^
Position designated	Percent of total	Percent of total
Defensive back	16.33	19.1
Defensive line	13.26	14.26
Kicker/punter	3.32	2.91
Linebacker	15.65	13.96
Offensive line	21.65	13.15
Quarterback	4.39	4.03
Running back	10.23	13.64
Tight end	6.74	5.97
Wide receiver	8.42	12.97

^a^ Actual number of responders minus those not in PFR database because of difference in criteria for selection into sample vs criteria for being in PFR (see text).

^b^ Assumes that all players completed Q1 in 2017.

^c^ Players with (last year‐first year = 0) given a value of 1.

Clearly, there are limitations to drawing definitive conclusions from what is essentially a voluntary participatory sample from the fully defined population of former players. These include issues of both sampling bias as well as generalizability. There are also issues of comparability of this cohort of essentially former super athletes to other men of comparable size, age, and race who were not as athletic. Thus, comparing the generalizability of the findings in these studies of former ASF players to the general population can only be done with caution. With regard to outcomes, we were able to show that the actual number of reported Anterior Cruciate Ligament (ACL) tears among our participating former players’ years of play^25^ corresponded almost exactly to that which would be predicted from data in the literature in two reports on ACL tears in active players summarizing the last 20 years.^26^, ^27^ In spite of this initial evidence of representativeness, any estimates of prevalence among our current responders can only be considered preliminary and used with caution. As we move forward with our 4‐year follow‐up assessment, we would anticipate our incidence data would become more generalizable within the total cohort.

One of the major objectives of initiating these studies was to define the nature of the risks these former players experience in their postplaying years, from the time they are no longer actively playing through the remainder of their lives. Too often, both the peer‐reviewed scientific literature and the lay press consists of anecdotal, unique, and often dramatic case reports. Even in the studies that have used collected samples from a series of cases, one cannot make estimates of the actual levels of risk. There would appear to be no question that there are potential long‐term health risks associated with participation in professional ASF, but the magnitude of these risks remains elusive. In addition, most published studies of health risks offer limited potential avenues for mitigation or prevention. The future health and well being of potential players both during the time they are playing as well as in their post play lives will be enhanced by developing a more quantitative understanding of the risks and possible benefits associated with life in football.

No less important in our studies is the possibility of understanding potential pathophysiologic mechanisms associated with or related to some of the variety of injuries and medical conditions associated with ASF. Because of the way the various studies described above have been undertaken, we have the opportunity to explore some of these mechanisms of injury and repair both in human and other animal species. In one case, a pilot/targeted study has led to a formal phase 3 clinical trial.^18^ One of our original cardiac pilot studies was instrumental to a multiyear NIH‐funded effort to examine mechanistic underpinnings of pathologic heart remodeling in football athletes. Two targeted studies designed to assess the potential for intervention for reducing chronic pain or hypertension are also underway. If newly designed brain contrast assessments in our IPA studies correlate with standardized neurocognitive testing, these efforts may provide a further understanding of the chronic repetitive head injury.

## 4 TURNING SCIENCE INTO ACTIONABLE INSIGHTS

Future studies linked to biological targets will hopefully yield quality of life improvements for players and former players, but predicting who might become affected, what specific exposures increase that risk, whether there are postplaying activities that will mitigate that risk, and whether, once affected, the condition can be treated or its impact minimized, remains to be determined. Even while waiting for definitive studies, there may be opportunities to demonstrate potentially important interventions. Several analyses on the initial data have resulted in publications or manuscripts related to domains of interest. For example, our data strongly suggest that voluntary or forced weight gain during active playing years increases downstream risk in a variety of domains, providing valuable information for player education and clinical practice.^28^ Similarly, a recent analysis of long‐term outcomes from having torn an ACL during active play suggests that, besides increased risks of significant arthritis and subsequent knee replacement, there is a need for assessing former players for potential cardiac risks, as there appears to be a modest excess of myocardial infarction among these former players.^25^ These findings suggest that former players should undergo cardiovascular risk assessment, and that certainly for those with post ACL injuries and resulting chronic knee pathology, or chronic significant weight gain during playing years, consideration of custom designed exercise programs is warranted. Evaluating the impact of providing specific information in a focused form to former players is an obvious and important component that is being developed as part of our long‐term follow‐up plans.

## 5 CONCLUSIONS

The Football Players Health Study at Harvard University is a broad and ambitious research and translation program that attempts to securely capture data from all aspects of former ASF players’ lives. We anticipate that such data will help to quantify the potential long‐term risks associated with ASF. As more pathophysiologic data and risk quantification are obtained, we anticipate the information will be useful to drive more informed player decision‐making. We would also anticipate that results will lead to appropriate interventions as these men age, and thus enhanced health and wellbeing outcomes.

## CONFLICT OF INTERESTS

Dr. Zafonte was partially supported by the National Institute on Disability, Independent Living, and Rehabilitation Research (90DP0039‐03‐00, 90SI5007‐02‐04, and 90DP0060), the National Institutes of Health (4U01NS086090‐04, 5R24HD082302‐02, and 5U01NS091951‐03), and US Army Medical Research and Materiel Command (W81XWH‐112‐0210). He also serves as Co‐PI on a T‐32 and receives funding from the Football Players Health Study at Harvard University, which is funded by the NFL Players Association. Dr. Zafonte received royalties from (a) Oakstone for an educational CD—Physical Medicine and Rehabilitation: a Comprehensive Review; (b) Demos publishing for serving as coeditor of the text Brain Injury Medicine. Dr Zafonte serves on the Scientific Advisory Board of Myomo, Oxeia Biopharma, BioDirection, and ElMINDA. He also evaluates patients in the MGH Brain and Body—TRUST Program, which is funded by the NFL Players Association.

Dr. A. Pascual‐Leone was partly supported by the Sidney R. Baer Jr. Foundation, the National Institutes of Health (R01MH100186, R21AG051846, R01MH111875, R01MH115949, R01MH117063, R24AG06142, and P01 AG031720), the National Science Foundation, DARPA, the Football Players Health Study at Harvard University, and Harvard Catalyst| The Harvard Clinical and Translational Science Center (NCRR and the NCATS NIH, UL1 RR025758). Dr. A. Pascual‐Leone serves on the scientific advisory boards for Neosync, Neuronix, Starlab Neuroscience, Neuroelectrics, Magstim Inc, Constant Therapy, and Cognito; and is listed as an inventor on several issued and pending patents on the real‐time integration of transcranial magnetic stimulation with electroencephalography and magnetic resonance imaging.

Dr. Baggish has received funding from the National Institutes of Health/National Heart, Lung, and Blood Institute, the National Football League Players Association, the American Heart Association, the American Society of Echocardiography and receives compensation for his role as team cardiologist from US Soccer, US Rowing, the New England Patriots, the Boston Bruins, the New England Revolution, and Harvard University.

All authors in this study are or were either partially or fully supported by the Football Players Health Study at Harvard University which is in turn sponsored by the NFLPA.

## AUTHOR CONTRIBUTIONS

All authors participated in the conception and design of the study, acquisition, analysis, and interpretation of the data, participated in drafting and revising the manuscript and approved the final version submitted to AJIM. All authors agreed to be accountable for all aspects of the study to ensure that questions related to its accuracy and integrity are appropriately investigated and resolved.

## ETHICS APPROVAL AND INFORMED CONSENT

The work was performed at Harvard Medical School. The institutional review board of the Beth Israel Deaconess Medical Center, Boston, USA approved this study and all participants provided written consent before participating in the research. We secured a Certificate of Confidentially from the National Institute of Health for each individual research protocol developed which includes former player participants. The Certificate prohibits disclosure of identifiable, sensitive research information to anyone not connected to the research (with certain exceptions; see https://humansubjects.nih.gov/coc/index).

## ENDNOTES

1 NFL Profootball Reference, an on‐line website that follows NFL player statistics both currently and historically.

2 Most of these sources overlapped with the main sources of identified contact information obtained from the NFL Players Association, and the NFL Alumni Association.

3 2008‐2012 PROMIS Health Organization and PROMIS Cooperative Group.

4 The applications was designed specifically for Apple iPhones and participants were invited to access many functions that measured a number of physiologic parameters while, standing, walking, and dual tasking.
